# Meningiomas and Cognitive Impairment after Treatment: A Systematic and Narrative Review

**DOI:** 10.3390/cancers13081846

**Published:** 2021-04-13

**Authors:** Renato Gondar, Gildas Patet, Karl Schaller, Torstein R. Meling

**Affiliations:** 1 Neurosurgical Division, Department of Neurosciences, Geneva University Hospitals, 1206 Geneva, Switzerland; rjag20@gmail.com (R.G.); gildas.patet@hcuge.ch (G.P.); Karl.Schaller@hcuge.ch (K.S.); 2 Faculty of Medicine, University of Geneva, 1206 Geneva, Switzerland

**Keywords:** meningioma, cognition, neuropsychology, language, memory, attention, executive function, prognosis

## Abstract

**Simple Summary:**

Assessment of cognition is crucial in brain tumor care, and clinical outcome along this axis is frequently neglected. As a result, a patient’s quality of life seems more impacted than usually reported in clinical series. With this article, we review the current state of affairs and search for patient- and meningioma-related outcome predictors. We found a great variety in the number and types of neuropsychological tests used and in the dimensions of cognition studied. Furthermore, data mostly originate from a selected part of the globe and therefore may not reflect a global reality. Treatment has an early cognitive impact in the majority of meningioma patients. Further long-term conclusions are precluded by a mean follow-up time shorter than one year. Anticipating cognition outcomes prior to, during, and after treatment of meningiomas remains difficult. Future research should aim for a reliable and worldwide reproducible standard battery of tests.

**Abstract:**

Clinical outcomes after surgery for intracranial meningiomas might be overvalued as cognitive dimensions and quality of life are probably underreported. This review aims to summarize the current state of cognitive screening and treatment-related outcomes after meningioma surgery. We present a systematic review (Preferred Reporting Items for Systematic reviews and Meta-Analyses (PRISMA-P) 2015-based) of cognitive outcomes after intracranial meningioma surgery. A total of 1572 patients (range 9–261) with a mean age of 58.4 years (range 23–87), and predominantly female (*n* = 1084, 68.9%) were identified. Mean follow-up time after treatment was 0.86 ± 0.3 years. Neuropsychological assessment was very heterogeneous, but five dimensions of cognition were described: memory (19/22); attention (18/22); executive functions (17/22); language (11/22); flexibility (11/22 studies). Cognitive abilities were impaired in 18 studies (81.8%), but only 1 showed deterioration in all dimensions simultaneously. Memory was the most affected. with significant post-therapy impairment in 9 studies (40.9%). Postoperatively, only 4 studies (18.2%) showed improvement in at least one dimension. Meningioma patients had significantly lower cognitive scores when compared to healthy subjects. Surgery and radiotherapy for meningiomas were associated with cognitive impairment, probably followed by a partial recovery. Cognition is poorly defined, and the assessment tools employed lack standardization. Cognitive impairment is probably underreported in meningioma patients.

## 1. Introduction

T Intracranial meningiomas (ICMs) account for 30% to 40% of all primary tumors of the central nervous system (CNS) [1,2]. Whereas microsurgical resection is the gold standard for the treatment of ICMs with documented growth on serial imaging or with symptoms referable to the lesion, refractory to medical therapy and significant oedema, radiotherapy or radiosurgery is considered for patients who are not surgical candidates, for some deep inaccessible tumors, or for atypical or malignant meningiomas either after initial subtotal resection or after recurrence.

Technical advances in micro-neurosurgery and radiotherapy have allowed increasingly effective meningioma management with respect to survival and morbidity [1,2,3,4]. However, clinical outcomes after surgery for meningiomas might be overvalued as cognitive dimensions and quality of life are probably underreported. Despite advances in tools that allow intra-operative neuromonitoring of sensory, motor and speech functions, neurosurgeons remain unable to maintain an online control of other cognitive functions during brain tumor resections, and a large proportion of patients still suffer treatment-induced cognitive deficits that dramatically reduce their life quality [5,6,7,8,9,10]. Therefore, assessment of health-related quality of life (HRQoL) is an important outcome measure in brain tumor research and management [5,6,7,8,9,10].

Among HRQoL measures, cognitive function through its different dimensions, as well as disease-related anxiety or depression, should be routinely assessed [5,6,7,8,9]. Cognition, as defined by Coltheart in 2001 [11], represents the study of cognitive processes through data from people who suffer from either developmental or acquired disorders of cognition. From a somatic point of view, this definition is challenged by the dichotomy between neuroanatomy and physiology, taking into account localizationist (Broca, 1861) [12] and connectionist (Abel, 2007) models [13]. Furthermore, the implications of neurological impairments depend on the patient’s profession, daily activities, and lesion laterality and location. To address the latter, recent studies have focused beyond anatomical lobar or hemispherical boundaries and tried to correlate white matter tracts and different brain networks’ disruption with cognitive impairment prediction [14,15,16,17,18].

With this review, we aim to summarize the state of affairs in terms of neuropsychological and cognitive screening, both pre- and postoperatively or after radiation therapy, for patients with meningiomas. At the same time, we aim to identify patient- and tumor-dependent predictors of treatment-related cognitive impairment in terms of tumor location (lobar, hemispheric or white matter invasion or compression), patient age, tumor size, peritumoral oedema, histological grade, and extent of resection. 

## 2. Materials and Methods

### 2.1. Search Strategy and Study Selection

Our retrospective work was conducted according to the Preferred Reporting Items for Systematic reviews and Meta-Analyses (PRISMA-P) 2015 guidelines [19].

A combination of the keywords (meningioma AND cognition) OR (meningioma AND neuropsychology) OR (meningioma AND language) OR (meningioma AND memory) OR (meningioma AND attention) allowed a targeted search made on 15th November 2020 on the following databases: Embase, Cochrane Library, PubMed, Google Scholar, and Web of Science.

**Figure 1:**
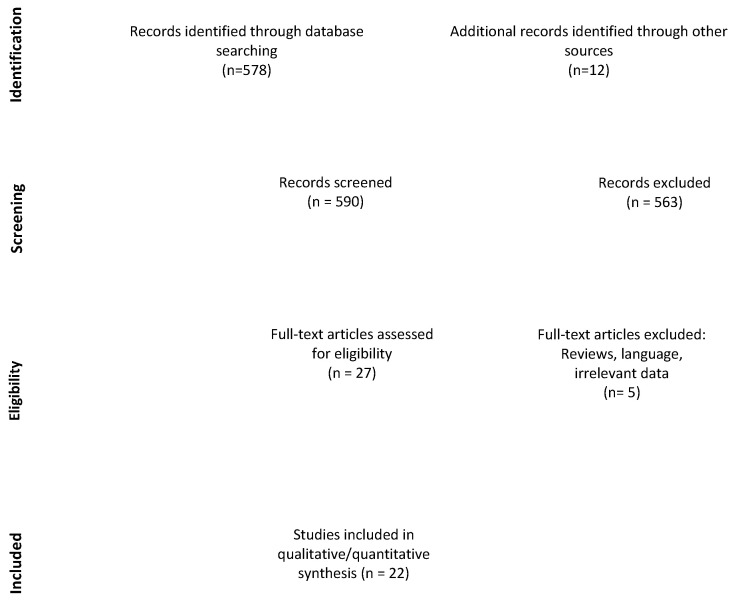
Preferred Reporting Items for Systematic reviews and Meta-Analyses 2015 (PRISMA-P) flow-chart and search strategy.

The final list of 578 articles was completed with 12 additional studies found among their corresponding references. Two authors (RG and GP) independently screened titles and abstracts of all identified articles, and full-text copies of all relevant articles were acquired. In the case of a discrepancy, the senior author (TRM) would arbitrate until a consensus among the authors was reached (Figure 1). 

The following inclusion criteria were used: (1) peer-reviewed research articles, prospective or retrospective, on cognitive functioning in adult patients with meningioma prior to and following surgery with or without adjuvant radiotherapy or radiosurgery, as assessed with neuropsychological tests; (2) samples of at least 5 meningioma patients included; (3) studies written in the English, French, German, or Portuguese language; (4) results of studies that examined cognition in brain tumor patients were also included if separate analyses were done for meningioma patients.

Studies that used very short screening tests, such as Mini-Mental State Examination (MMSE) and 3MS examination (modified MMSE), or that only used self-reported patient-reported outcome measurements (PROMs) and physician-reported measures were also excluded. Additionally, editorials, letters, review articles, and case reports were not included.

The last step of the systematic review process involved thorough reading of 27 articles, of which 1 article did not present enough data to meet the inclusion criteria and 4 articles failed to compare the cognitive results prior to and after treatment. Therefore, 22 articles were included in our final analysis [5,20,21,22,23,24,25,26,27,28,29,30,31,32,33,34,35,36,37,38,39,40]. After noticing that some articles came from the same institution, and after confirming with a corresponding author, we decided not to perform a meta-analysis because of bias induced by duplicates between studies. 

### 2.2. Risk of Bias and Quality of Study

The accepted articles were independently graded by two authors (RG and GP) according to the Newcastle–Ottawa Quality Assessment Scale for quality assessment of non-randomized studies [41]. All the included studies were quoted as fair or good quality. The level of evidence for each study was evaluated using the Oxford Centre for Evidence- Based Medicine guidelines [42].

### 2.3. Data Collection

The first two authors (RG and GP) independently extracted the data from the included studies. The following data items were considered: (1) study ID; (2) study characteristics (author, year, country, prospective or retrospective study); (3) patient demographics; (4) definition of cognitive impairment; (5) sample size; (6) meningioma location, size, and treatment; (7) cognition measurement tools; 8) cognition at baseline and at follow-up; (9) brain invasion from meningioma; (10) follow-up (FU) time; (11) other PROMs; (12) parameters concerning intra- and postoperative complications (complications, estimated blood loss, and duration of surgery). If necessary, consensus was reached by both authors through discussions with the senior and last author (TRM). 

### 2.4. Statistical Analysis

Results for continuous variables are reported as mean ± standard deviation (SD) or range. For articles that did not report mean and SD, we estimated the mean and SD according to the methodology described by Hozo et al. [43]. Categorical variables are presented as median and quartiles or by absolute and relative frequencies.

## 3. Results

### 3.1. Demographic Results

Table 1 summarizes the characteristics of all included studies and their corresponding patients that underwent neuropsychological cognitive evaluation prior to and after treatment. Overall, 9 retrospective [5,23,24,30,33,34,35,36,37] and 13 prospective [20,21,22,25,26,27,28,29,31,32,38,39,40] studies were reviewed, corresponding to a total of 1572 patients (range 9–261) with a mean age of 58.4 years (range 23–87). Most patients were female (n = 1084, 68.9%), with a female-to-male ratio of 2.3:1.

With regard to patients’ background, a mean education of 10.3 ± 2.9 years or a 4th to 5th level scholarity were described among studies, but only half of the studies provided these data (Table 1 and Table 2 and Supplementary Materials).

The meningioma location was not uniformly reported among groups, varying from a distinction between left- or right-sided only (31.5% vs. 33.1%) in 15 out of 22 papers or a more detailed anatomical (lobar; convexity; skull base; implantation basis) division for the rest (7/22 papers). The neuropsychological assessment postoperatively was performed at a mean of 0.86 ± 0.3 years after surgery.

### 3.2. Definition of Cognition

An important source of potential bias is the nomenclature and definition of cognition used by each research group. This fact leads each of the authors to potentially evaluate slightly different outcomes. Ultimately, the subsequent comparison of the studies can be inexact and the differences in subsequent results uninterpretable. Among the articles, cognition is loosely defined as the ability to name, recall, plan, and execute (Table 2 and Supplementary Materials). Others focus on memory only or quality of life and Karnofsky performance status (Table 2 and Supplementary Materials). Lastly, some authors also make reference to the capacity or personal adjustment and emotional function, as well as adaptative function. Even emotional behavior and anxiety or sleep-related issues are mentioned in this large notion of cognition.

### 3.3. Dimensions of Cognition and Respective Assessment Tools

The heterogeneity in definitions of cognition reflects itself in the variety of assessment tools and questionnaires used. After revision of the list of included articles, we were able to categorize cognition in the following five dimensions: verbal, working and visual memory (19/22 studies); Complex attention and orientation (18/22 studies); Executive functioning (17/22 studies); Language and verbal fluency (11/22 studies); Cognitive flexibility (11/22 studies) (Table 1).

Several measuring instruments aiming an objective neurocognitive description have been described (Table 1 and Table 2 and Supplementary Materials). Among the most common and reproducible ones, the Central Nervous System Vital Signs test (CNSVS) [44] (10/22 studies) comprises seven tests: verbal and visual memory, finger tapping, symbol digit coding, the Stroop Test, a test of shifting attention, and the continuous performance test. Secondly, the Hopkins Verbal Learning Test—Revised (HVLT-R) [45] consists of three domains, i.e., Total Recall (TR), Delayed Recall (DR), and Delayed Recognition (DRec), which are respectively related to immediate and learning memory, delayed memory, and recognition. Thirdly, the European Organization for Research and Treatment of Cancer Quality of Life Questionnaire Core 30 (EORTC QLQ-C30) [45] is composed of one scale measuring an individual’s global health status (GHS), five functional scales (physical, role, social, emotional, and cognitive functioning), and nine symptom scales (fatigue, nausea/vomiting, pain, dyspnea, insomnia, appetite loss, constipation, diarrhea, and financial difficulties). Fourthly, the Quality of Life Questionnaire 20 (QLQ-BN20) [45] (3/22 studies) is a disease-specific module for brain cancer patients, and it consists of 11 symptom scales: future uncertainty, visual disorder, motor dysfunction, communication deficit, headaches, seizures, drowsiness, itchy skin, hair loss, weakness of legs, and bladder control. In addition, the Raven Matrices, Objects and Verbs naming, ideomotor apraxia, and Token Test are only referred to in 2/22 studies. Lastly, the Benton test (orientation and attention, perception, memory, verbal functions and language skills, construction and motor skills, concept formation and reasoning and executive functions) and other neuropsychological tests such as Trail-Making Test (TMT), and the Control Oral Word Association Test (COWAT) that assesses semantic fluency, were used in 7/22 studies [44,46].

### 3.4. Outcome: Consequences of Therapy

The identified literature consists of 18 cohorts treated with surgery [5,20,21,22,23,24,25,26,27,28,29,30,31,32,33,34,36,40], 3 cohorts that benefited from both surgery and adjuvant radiotherapy due to partial tumor removal [35,37,39], and 1 cohort that only received radiotherapy/radiosurgery [38] as a single treatment modality (Table 2 and Supplementary Materials). Table 2 provides a selected subset of the 5 biggest cohorts analyzed. The entire description of the 22 cohorts can be found online as a Supplementary Table.

Overall results show that cognitive function declined post-therapy in most cases (n = 18 studies, 81.8%) in several of the previously discussed five domains (Table 1). Only one study showed a clear aggravation in all domains at the same time. Verbal, working, and visual memory were the most frequently affected dimensions, with significant post-therapy impairment in nine studies (40.9%). The lesser studied was language and verbal fluency (only two studies, or 9.1%). On the other hand, four studies (18.2%) showed an improvement in at least one dimension without concomitant impairment. This positive effect was mostly noticed in verbal, working, and visual memory and complex attention and orientation (*n* = 6, 27.3%).

Some, but not all, studies compared the results with healthy populations and concluded that meningioma patients had significantly lower scores in several cognitive domains, most frequently in cognitive flexibility (*n* = 4, 18.2%), despite experiencing improvements after surgery (Tucha et al.) [40] or fractioned stereotactic radiotherapy (Steinvorth et al.) [38].

Comparisons between patients treated with surgery only and patients treated with surgery and adjuvant radiotherapy [33,37,39] showed no significant difference in cognition scores attributable to the radiotherapy itself, although no matching was performed for tumor- and patient-characteristics. Therefore, no evidence was found that additional radiotherapy negatively impacted cognition. One must point out that two of these studies failed to provide clear pre-treatment assessment for most of the evaluated dimensions.

Finally, another potential source of bias lies in the fact that some of the available studies have evaluated a very low number of patients (<25 in some cases). By doing so, the risk of a type II statistical error increases.

## 4. Discussion

This focused systematic review describes the current state with respect to cognition and meningioma treatment, and we identified several interesting features (Table 1). The first is that cognition is scarcely studied in the context of meningiomas, with very few studies identified and even fewer centers involved in publishing such data, most of those from Northern Europe. This indicates that cognitive impairment could be underreported and also precludes worldwide extrapolation of the results. Secondly, even though meningiomas are extra-axial lesions, they usually become symptomatic due to a mass effect that subsequently impacts different dimensions of cognition, depending on tumor location. Thirdly, as detailed in Table 2 and the Supplementary Materials, the studies generally concluded that cognitive impairment after surgery and/or radiotherapy is frequent (81.8%) [5,20,21,22,23,24,25,26,27,28,29,30,31,32,33,34,35,36,37,38,39,40], particularly with respect to different memory modalities (40.9%). This observation was made more frequently when pre- and postoperative assessments were compared than when patients were compared to a healthy general population (Table 1). Postoperatively, verbal deficits were more pronounced if the tumor was located on the left side (1 study) [20]; subjective functioning, anxiety, and depression worsened (3 studies) [21,31,33]; working memory deterioration was more frequent in left-sided lesions compared to controls (1 study) [36]; and there was a significant correlation between pre-operative tumor volume and postoperative cognitive functioning (1 study) [23]. Lastly, a few teams were able to conduct follow-ups at one year after intervention and observed an incomplete recovery of cognition after an initial decline (Table 2 and Supplementary Materials).

There were important methodological and statistical issues that can bias our conclusions, as different authors employed different assessment tools (some giving too much importance to self-reported PROMs (EORTC QLQ-C30 and BN-20) and physician-reported KPS(Karnofsky Performance Status)), and not all assessed the five dimensions of cognition, not to mention the small size of some cohorts (nine below 50 patients and six below 25) (Table 2 and Supplementary Materials). Furthermore, the definition of cognition also varied widely and was difficult to standardize. The current literature generally categorizes cognitive neuropsychology into five dimensions: memory, attention and orientation, executive capacities, language, and task flexibility. Along these dimensions, a wide variety of tests, scales, questionnaires, and scores try to objectively quantify an eventual deficit, and this sometimes becomes redundant. These tools also depend on patient-related characteristics, such as education, which in turn are also evaluated through different scales. Cultural, social, and scholarly backgrounds influence the baseline cognitive performance of our patients. This is of paramount importance when patients are compared to healthy controls. In order to make any meaningful evaluation of the impact on the quality of life for each meningioma patient, it would be necessary to study the effective implications in patients’ professions and hobbies. Although it is understandably difficult to go into individual detail in these aspects, several authors state them as good indicators of quality of life [5,6,7,8,9,10,14]. Furthermore, there was important heterogeneity with respect to reporting of meningioma-related characteristics. Location was variable and frequently heterogeneously reported in most of the studies, making inferences regarding causality difficult (Table 2 and Supplementary Materials). Furthermore, there was scarce information regarding extent of resection, oedema, histology, or cortex invasion of the lesion. Indeed, some studies that reported such parameters focused more on the radiological side and failed to present simultaneous pre- and post-operative cognitive assessments. This issue led to their exclusion from this systematic review. Another structural limitation in most of the studies was the short follow-up time, as the mean duration was less than one year, probably as a consequence of the limited neuropsychologist resources in neurosurgical departments. However, for slow-growing tumors that are mostly benign, such as meningiomas, with minor impact on the patients’ average residual life expectancy [1,2,3,4], outcome assessment after less than one year is practically irrelevant and too soon to evaluate a potential positive effect of the treatment on cognition.

As a result of the heterogeneity exposed in the preceding paragraphs, firm conclusions and comparisons should be taken with caution, and current evidence fails to identify patient- and tumor-dependent predictors of treatment-related cognitive impairment. Consequently, we should work to standardize assessment tools and to establish more rigorous testing at defined intervals and with longer follow-up. This step could further lead to a better outcome prediction, subsequent identification of patients at higher risk for post-operative cognitive deficits, and possibly identify differences in outcome between treatment modalities, such as surgery or radiotherapy/radiosurgery. The balance between aggressive treatment and preservation of quality of life and neurological status continues to rely on subjective criteria and patient will and prior status.

This review also presents some limitations, mostly due to the diverse and few systematized approaches that this binomial pair, meningioma and cognition, has in the current literature and practice. Namely, the fact that multiple studies describe overlapping study populations prevents a solid meta-analysis. Nevertheless, their contribution to the narrative review and understanding of this topic is of major importance, and the tools and aspects studied are complementary between each other. The decision to describe cognition within HRQoL measurement is also debatable, but in our understanding, both fields are interdependent, as discussed in the Introduction chapter.

## 5. Conclusions

Both modalities of treatment, microsurgery and radiotherapy, are associated with cognitive impairment at an early stage, probably followed by a partial recovery, but patients tend to keep lower cognitive scores when compared with the healthy population.

Several factors, such as the complexity of systems responsible for cognitive functions and the ongoing study of the interactions between connectionist and localizationist brain structures, make the understanding and prediction of cognitive deficits very difficult. The presence of several limiting factors, such as lack of pre-treatment assessments, variations in the number and types of neuropsychological tests used and in the definition of cognitive impairment, and the quality of patient- and meningioma-related data, prevents us from drawing conclusions or cleaner comparisons among studies.

Further work should focus on identifying reliable and reproducible cognition assessment tools. Further, longer and regular evaluation intervals are needed in order to accurately evaluate cognition and quality of life in meningioma patients.

## Figures and Tables

**Table 1:** Summary of all studies on meningioma and cognition (study and patient characteristics). * according to the Dutch scoring system (Verhage), which consists of an eight-point scale, ranging from unfinished primary education (level 1) to university level (level 8).

Study Design	Retrospective: 9
	Prospective: 13
**Number of patients**	Total: 1572 (range: 9–261 per study)
	Mean: 71.5 per study
**Age**	Mean: 58.4 y
	Range: 23–87 y
**Gender**	Not stated: 25/1′572 (1.6%)
	Female: 1′084/1′572 (68.9%)
	Male: 463/1′572 (29.5%)
**Localization**	Not stated: 395/1′572 (25.1%)
	Left hemisphere: 495/1′572 (31.5%)
	Right hemisphere: 520/1′572 (33.1%)
	Bilateral: 162/1′572 (10.3%)
	Frontal lobe: 403/1′572 (25.6%)
**Mean education**	Not stated: 588/1′572 (37.4%)
	Mean in years: 10.3 ±2.9
	Mean in level: 4.6 *
**Assessment measures/tools**	Central Nervous System Vital Signs tests (10/22 studies)
	HVLT-R, EORTC, QLQ-30, and QLQ-BN20 (3/22 studies)
	Raven Matrices, Objects and Verbs naming, ideomotor apraxia, Token Test (2/22 studies)
	Other neuropsychological test (7/22 studies)
**Dimension of cognition tested**	Verbal, working and visual memory (19/22 studies)
	Complex attention and orientation (18/22 studies)
	Executive functioning (17/22 studies)
	Language and verbal fluency (11/22 studies)
	Cognitive flexibility (11/22 studies)
**Neuropsychological outcome** *Comparison prior to and after treatment*	Worsening of verbal, working and visual memory (9/22 studies)
	Worsening of complex attention and orientation (1/22 studies)
	Worsening of executive functioning (3/22 studies)
	Worsening of language and verbal fluency (2/22 studies)
	Worsening of cognitive flexibility (4/22 studies)
	Worsening in all neurocognitive domains (1/22 studies)
	Improvement in verbal, working and visual memory (3/22 studies)
	Improvement of complex attention and orientation (3/22 studies)
	Improvement of executive functioning (2/22 studies)
	Improvement of cognitive flexibility (1/22 studies)
*Comparison with healthy population*	Worse verbal, working and visual memory (2/22 studies)
	Worse complex attention and orientation (1/22 studies)
	Worse executive functioning (1/22 studies)
	Worse language and verbal fluency (2/22 studies)
	Worse cognitive flexibility (4/22 studies)
**Follow-up (years ± SD)**	Not stated: 4/22 studies (18.2%)
	Mean: 0.86 ± 0.3 years

**Table 2:** Detailed description of the 5 biggest cohorts (study and patient characteristics). Controlled Oral Word Association Test (COWAT); female (F), male (M); not acknowledged (n.a.); Rivermead Behavioral Memory Test (RBMT); standard deviation (SD); Trail making Test (TMT); years (y).

Author, Year, Design	Sample Size (n)	Gender	Mean Age (y ± SD, Range)	Mean Education (y ± SD or Level)	Location	Assessment Measurement Tools	Cognition Dimensions Tested	Difference Between Pre- and Post-Op or Against Control Group	Follow-Up (y)
**Van Lonkhuizen, 2019, prospective**	242	168 F & 74 M	57.2 (23–82)	5th level	101 Left and 113 Right; 142 Frontal and 100 Non-frontal	Central Nervous System Vital Signs tests	Verbal and visual memory, reaction time, complex attention, and cognitive flexibility	Worsening of SCF, anxiety and depression before and at 1y (*p* < 0.01) with surgery No difference after surgery	1
**Rijnen, 2019, prospective**	261	189 F & 72 M	57.8 ± 11.7 (23–82)	14.0 ± 3.7	106 Left, 124 Right and 31 Bilateral; 154 Frontal and 107 Non-frontal	Central Nervous System Vital Signs tests	Verbal and visual memory, reaction time, complex attention and cognitive flexibility	Worsening of verbal memory, visual memory, processing speed, psychomotor speed, reaction time, attention complex and cognitive flexibility with surgery (*p* < 0.05)	1
**Pranckevičienė, 2019, prospective**	93	68 F & 25 M	63.8 +/- 10.7	n.a	36 Left, 37 Right, and 20 Bilateral	HVLT-R, EORTC, QLQ-30 and QLQ-BN20	Verbal, working and visual memory and complex attention	Worsening of working memory, delayed recall and recognition, flatter learning slope and less effective acquisition compared to control group	n.a
**van der Vossen, 2014, retrospective**	136	106 F & 30 M	59.1 ± 12.7	n.a	66 Convexity and 70 Non-convexity	CFQ and HADS	Cognitive flexibility, anxiety, and depression	Worsening of cognitive and/or emotional problems in 40% of patients with surgery	3.0 ± 0.9
**Krupp, 2009, retrospective**	91	60 F & 31 M	56 ± 10 (31–75)	n.a	48 Left and 43 Right; 40 Frontal and 51 Non-frontal	Others	Language and cognitive flexibility	Worsening of concentration performance, verbal knowledge, technical ability, and word fluency compared to control group	1.25 ± 0.3

## Data Availability

Data sharing not applicable as no new data were created or analyzed in this study. Data sharing is not applicable to this article. However, data from the included articles are contained within the article or Supplementary Materials in Table 1 and Table 2, and the Supplementary Materials section.

## Supplementary Materials

The following are available online at https://www.mdpi.com/article/10.3390/cancers13081846/s1, Detailed description of each cohort (study and patient characteristics). Controlled Oral Word Association Test (COWAT); female (F), male (M); not acknowledged (n.a.); Rivermead Behavioral Memory Test (RBMT); standard deviation (SD); Trail-Making Test (TMT); years (y).
