# Supplementary material for: Meningiomas and Cognitive Impairment after Treatment: A Systematic and Narrative Review
Source: Cancers (Basel). 2021 Apr 13;13(8):1846. doi: 10.3390/cancers13081846 (PMC8070481; doi:10.3390/cancers13081846)
Supplement: Supplementary file 1 [file cancers-13-01846-s001.pdf]

Author, year, design	Sample size (n)	Gender	Mean age (y ± SD, range)	Mean education (y ± SD or level)	Location	Assessment measurement tools	Cognition dimensions tested	• Difference between pre- and post-op or against control group	Follow-up (y)
**Van Lonkhuizen, 2019, prospective**	242	168 F & 74 M	57.2 (23-82)	5th level	101 Left & 113 Right // 142 Frontal & 100 Non-frontal	Central Nervous System Vital Signs tests	Verbal and visual memory, reaction time, complex attention and cognitive flexibility	• Worsening of SCF, anxiety and depression before and at 1y ($p < 0.01$) with surgery<br>• No difference after surgery	1
**Rijnen, 2019, prospective**	261	189 F & 72 M	57.8 ± 11.7 (23-82)	14.0 ± 3.7	106 Left, 124 Right and 31 Bilateral // 154 Frontal and 107 Non-frontal	Central Nervous System Vital Signs tests	Verbal and visual memory, reaction time, complex attention and cognitive flexibility	• Worsening of verbal memory, visual memory, processing speed, psychomotor speed, reaction time, attention complex and cognitive flexibility with surgery ($p<0.05$)	1
**Van Nieuwenhuizen, 2019, retrospective**	21	14 F & 7 M	55.3 (10.6)	4.0 (1.8) level	13 Convexity, 5 Skull base & 3 Orbital	Central Nervous System Vital Signs tests	Verbal and working memory, attention, executive functioning and cognitive flexibility	• Significant correlation between preoperative cerebral edema and tumor volume and postoperative cognitive functioning	0.4
**Pranckevičienė, 2019, prospective**	93	68 F & 25 M	63.8 +/- 10.7	n.a	36 Left, 37 Right and 20 Bilateral	HVLT-R, EORTC, QLQ-30 and QLQ-BN20	Verbal, working and visual memory and complex attention	• Worsening of Working memory, Delayed recall and recognition, Flatter learning slope and less effective acquisition compared to control group	n.a
**Van Nieuwenhuizen, 2018, retrospective**	20	14 F & 6 M	51.4 (10.9)	4 (6) level	12 Frontal, 4 Parietal, 2 Temporal and 2 Occipital	Central Nervous System Vital Signs tests	Verbal and working memory, attention and executive functioning	• None	n.a

**Di Cristofori, 2018, prospective**	41	15 F & 26 M	74 (70–87)	n.a	7 Skull base, 9 Falx cerebri, 7 Posterior fossa and 18 Convexity	Raven Matrices, Objects and Verbs naming, ideomotor apraxia, Token Test, among others	Verbal and working memory, attention, executive functioning, language	• Improvement in Language, memory, attention, executive verbal functions, executive non-verbal functions and praxis with surgery ($p<0.01$)	1
**Zweckberger, 2017, prospective**	58	47 F & 11 M	56.4 ± 12.5	n.a	46 Anterior skull base, 5 Middle and 7 Posterior fossa	HVLT-R, EORTC, QLQ-30 and QLQ-BN20	Verbal, working and visual memory, attention, executive functioning, language	• Improvement of Verbal learning, visual learning memory, Visuomotor processing speed and working memory ($p<0.05$) with surgery	1
**Hendrix, 2017, prospective**	12	7 F & 5 M	62.0 ± 13.2	n.a	1 Left, 5 Right and 6 Bilateral	Central Nervous System Vital Signs tests	Verbal and working memory, attention, executive functioning, language	• Worsening of Verbal fluency and Perceptual speed compared to control group	0.17
**Abel, 2016, retrospective**	70	45 F & 25 M	62.1 ± 12.6	13.5 ± 2.5	23 ventromedial & 47 non-ventromedial prefrontal cortex	Others	Verbal and visual memory, attention and orientation, executive functioning, language skills, cognitive flexibility	• Worsening of behavior and specifically adaptive functions with surgery	n.a
**Liouta, 2016, prospective**	54	38 F & 16 M	56.8 ± 12.8	10.2 ± 2.4	17 Left, 27 Right and 10 Bilateral // 28 Convexity and 26 Skull base	Wechsler Adult Intelligence Scale-III Digit Span, TMT Parts A and B,	Memory, executive functioning, language and cognitive flexibility	• Verbal deficits more pronounced in the left than right hemisphere<br>• No deterioration in neurocognitive function after surgery	1

						COWAT, RBMT			
**Bommakanti, 2016, prospective**	57	35 F & 22 M	44.72 (17-64)	n.a	20 Left, 20 Right and 17 Bilateral	Others	Verbal, working and visual memory, attention and executive functioning	• Worst Memory, attention and executive functions (p: n.a) compared to control group	0.25
**Campanella, 2015, prospective**	16	n.a	57.94 ± 11.08	10.81 ± 4.02	9 Left & 7 Right; 6 Frontal, 8 Parietal and 2 Temporal	Central Nervous System Vital Signs tests	Attention and orientation, executive functioning, language and perception.	• Worsening of Alexithymia and Self-maturity with the surgery	0.33
**Meskal, 2015, prospective**	68	46 F & 22 M	55.66 (36–74)	4.90 (4–7) level	30 Left, 33 Right and 5 Bilateral; 39 Frontal & 29 Non-frontal	Central Nervous System Vital Signs tests	Memory, attention, processing speed, executive functioning and cognitive flexibility,	• Improvement memory, complex attention, cognitive flexibility, processing speed, and executive functioning ($p < 0.05$) with the surgery	0.25
**van der Vossen, 2014, retrospective**	136	106 F & 30 M	59.1 ± 12.7	n.a	66 Convexity and 70 Non-convexity	CFQ and HADS	Cognitive flexibility, anxiety and depression	• Worsening of cognitive and/or emotional problems in 40% of patients with surgery	3.0 ± 0.9
**Waagemans, 2011, retrospective**	89	66 F & 23 M	58.4 ± 13.2	3.8 (2.1) level	37 Left, 25 Right & 27 Bilateral; 45 Convexity & 44 Non-convexity	Others	Working and verbal memory, attention, executive functioning, language and cognitive flexibility	• Worsening of executive functioning, psychomotor speed, verbal and working memory and information processing capacity ($p< 0.05$) compared to control group	1
**Krupp, 2009, retrospective**	91	60 F & 31 M	56 ± 10 (31–75)	n.a	48 Left & 43 Right; 40 Frontal & 51 Non-frontal	Others	Language and cognitive flexibility	• Worsening of concentration performance, verbal knowledge, technical ability and word fluency compared to control group	1.25 ± 0.3

**Dijkstra, 2009, retrospective**	89	66 F & 23 M	58.6 ± 12.1	3.8 ± 2.2	37 Left, 25 Right and 27 Bilateral	Others	Executive functioning, Psychomotor speed, Working memory, Attention, Information processing and Verbal memory	• Worsening of executive functioning, verbal memory, information processing capacity, psychomotor speed and working memory ($p<0.05$) compared to control group	1
**Yoshii, 2008, retrospective**	9	n.a	61.8	n.a	4 Left and 5 Right	Central Nervous System Vital Signs tests	Language, attention, executive functioning, memory, and perception	• Worsening of working memory in left side compared to control group	n.a
**Van Nieuwenhuizen, 2007, retrospective**	18	15 F & 3 M	62.6 ± 11.8	n.a	n.a	HVLT-R, EORTC, QLQ-30 and QLQ-BN20	Verbal, working and visual memo, complex attention and language	• Worsening of memory compared to control group ($p < 0.05$)	1
**Steinvorth, 2003, prospective**	40	27 F & 13 M	55 ± 14	n.a	13 Left, 22 Right and 2 Bilateral	Others	Memory and attention	• Improvement of attention and memory functions with radiotherapy	1
**Tucha, 2003, prospective**	54	37 F & 17 M	57.8 ± 1.5	9.6 ± 0.2	22 Left, 21 Right and 11 Bilateral	Central Nervous System Vital Signs tests	Verbal and visual memory, psychomotor speed, reaction time, complex attention and cognitive flexibility	• Worsening of working memory and improvement of attentional functions with surgery	0-33 - 0-75
**Tucha, 2001, prospective**	33	21 F & 12 M	72.8 ± 0.9	n.a	14 Left, 13 Right & 6 Bilateral; 10 Frontal & 20 Non-frontals	Central Nervous System Vital Signs tests	verbal and visual memory, complex attention, language and cognitive flexibility	• Improvement of attention and memory functions with surgery<br>• Worsening of verbal and figural working memory compared to control group	0.21 - 0.41

Supplementary Table. Detailed description of each cohort (study and patient characteristics). COWAT (Controlled Oral Word Association Test); F (females), M (males); n.a. (not acknowledged); RBMT (Rivermead Behavioural Memory Test); SD (standard deviation); TMT (Trail making Test); y(years).
